# Data-Driven Approaches in Antimicrobial Resistance: Machine Learning Solutions

**DOI:** 10.3390/antibiotics13111052

**Published:** 2024-11-06

**Authors:** Aikaterini Sakagianni, Christina Koufopoulou, Petros Koufopoulos, Sofia Kalantzi, Nikolaos Theodorakis, Maria Nikolaou, Evgenia Paxinou, Dimitris Kalles, Vassilios S. Verykios, Pavlos Myrianthefs, Georgios Feretzakis

**Affiliations:** 1 Intensive Care Unit, Sismanogelio General Hospital, 37 Sismanogleiou Str., 15126 Marousi, Greece; sakagianni@sismanoglio.gr; 2 Anesthesiology Department, Aretaieio University Hospital, National and Kapodistrian University of Athens, Vass. Sofias 76, 11528 Athens, Greece; ckoufopoulou@uoa.gr; 3 Department of Internal Medicine, Sismanogleio General Hospital, 15126 Marousi, Greece; peterkouf13@gmail.com; 4 Department of Internal Medicine & 65+ Clinic, Amalia Fleming General Hospital, 14, 25th Martiou Str., 15127 Athens, Greece; s.kalantzi@flemig-hospital.gr; 5 Department of Cardiology & 65+ Clinic, Amalia Fleming General Hospital, 14, 25th Martiou Str., 15127 Athens, Greece; n.theodorakis@flemig-hospital.gr (N.T.); m.nikolaou@flemig-hospital.gr (M.N.); 6 School of Science and Technology, Hellenic Open University, 18 Aristotelous Str., 26335 Patras, Greece; paxinou.evgenia@ac.eap.gr (E.P.); kalles@eap.gr (D.K.); verykios@eap.gr (V.S.V.); 7 Faculty of Nursing, School of Health Sciences, National and Kapodistrian University of Athens, 11527 Athens, Greece; pmiriant@nurs.uoa.gr

**Keywords:** antimicrobial resistance, machine learning, k-means clustering, principal component analysis, genomic data analysis

## Abstract

**Background/Objectives:** The emergence of antimicrobial resistance (AMR) due to the misuse and overuse of antibiotics has become a critical threat to global public health. There is a dire need to forecast AMR to understand the underlying mechanisms of resistance for the development of effective interventions. This paper explores the capability of machine learning (ML) methods, particularly unsupervised learning methods, to enhance the understanding and prediction of AMR. It aims to determine the patterns from AMR gene data that are clinically relevant and, in public health, capable of informing strategies. **Methods:** We analyzed AMR gene data in the PanRes dataset by applying unsupervised learning techniques, namely K-means clustering and Principal Component Analysis (PCA). These techniques were applied to identify clusters based on gene length and distribution according to resistance class, offering insights into the resistance genes’ structural and functional properties. Data preprocessing, such as filtering and normalization, was conducted prior to applying machine learning methods to ensure consistency and accuracy. Our methodology included the preprocessing of data and reduction of dimensionality to ensure that our models were both accurate and interpretable. **Results:** The unsupervised learning models highlighted distinct clusters of AMR genes, with significant patterns in gene length, including their associated resistance classes. Further dimensionality reduction by PCA allows for clearer visualizations of relationships among gene groupings. These patterns provide novel insights into the potential mechanisms of resistance, particularly the role of gene length in different resistance pathways. **Conclusions:** This study demonstrates the potential of ML, specifically unsupervised approaches, to enhance the understanding of AMR. The identified patterns in resistance genes could support clinical decision-making and inform public health interventions. However, challenges remain, particularly in integrating genomic data and ensuring model interpretability. Further research is needed to advance ML applications in AMR prediction and management.

## 1. Introduction

Antimicrobial resistance has emerged as one of the most pressing global health challenges of the 21st century. The rapid proliferation of resistant pathogens undermines the efficacy of antibiotics, antivirals, antifungals, and antiparasitic agents, leading to increased morbidity, mortality, and healthcare costs worldwide [1]. According to the Review on Antimicrobial Resistance chaired by Jim O’Neill, AMR is projected to cause 10 million deaths annually by 2050 if current trends continue, surpassing cancer as a leading cause of death [2]. The economic impact is equally alarming, with estimates suggesting a cumulative cost to global economic output of up to US$100 trillion by 2050 [2].

The overuse and misuse of antimicrobial agents in human medicine, agriculture, and animal husbandry have accelerated the evolution of resistant strains [3]. Inappropriate prescribing, self-medication, and inadequate infection control practices contribute to the selection pressure that drives resistance [4]. The mobility of resistance genes via horizontal gene transfer further exacerbates the problem, enabling rapid dissemination across bacterial populations and geographical boundaries [5]. This genetic exchange may also be mediated through mechanisms such as conjugation, transformation, and transduction, hence allowing the resistance to disseminate even across distantly related species [6].

Most of the traditional AMR surveillance approaches are reactive, strenuous, and have poor scalability for the global scope of this problem [7]. These conventionally include phenotypic testing of isolated strains, which may be time-consuming and limit the detection of emerging resistance genes in non-culturable organisms [8]. There is a pressing need for innovative, data-driven approaches that will significantly improve the monitoring, prediction, and management of AMR.

Advances in genomic technologies have created an explosion of biological data that offers unparalleled opportunities to investigate the mechanisms and spread of resistance at a molecular level [9]. Millions of genomic data have been generated by high-throughput sequencing and metagenomic analyses from clinical isolates, environmental samples, and microbiomes [10]. The analysis of genetic material recovered directly from environmental samples through metagenomics will avoid the need for culturing [11]. However, meaningful insights from this complex and voluminous data present significant analytical challenges due to heterogeneity, noise, and high dimensionality of data [12].

While traditional regression models have been widely used in predicting AMR, they are often limited in their ability to handle large, high-dimensional datasets. ML algorithms, on the other hand, are more scalable and capable of uncovering hidden patterns in the data without requiring explicit predefined relationships between variables. This makes them particularly suited to analyzing genomic data and identifying novel resistance mechanisms. Machine learning, considered a subset of artificial intelligence, offers powerful tools for analyzing large-scale datasets and uncovering patterns not easily identifiable through traditional statistical methods alone [13]. In the context of AMR, ML methods have been applied to predict resistance phenotypes from genomic data, identify novel resistance genes, and model the spread of resistance within and between populations [14,15]. Both supervised and unsupervised approaches hold promise in enhancing our understanding of AMR and informing public health interventions [16,17].

Genomic features coupled with random forests, support vector machines, and neural networks also formed the basis for various supervised learning model applications for the prediction of antibiotic susceptibility [18]. For example, studies have illustrated the possibility of directly predicting minimum inhibitory concentrations (MICs) and resistance phenotypes directly from whole-genome sequencing data [19]. However, these models require labeled datasets with known resistance outcomes and, for many applications, may not exist or be comprehensive.

Unsupervised learning methods, however, do not make use of predefined labels but instead find intrinsic structures of the data [20]. In this respect, certain techniques, such as clustering and dimensionality reduction, are capable of showing relationships between genes, classes of resistance, and other features that participate in AMR. Using clustering algorithms, for instance, genes or organisms can be grouped by computing their similarity measures to find new mechanisms of resistance or transmission [21]. Feature extraction methodologies such as Principal Component Analysis (PCA) help in the visualization of high-dimensional data and ensure that only the most informative features are selected [22].

In this study, we have applied unsupervised ML techniques, such as K-means clustering and PCA, to identify patterns in the data of AMR genes. Our analysis is based on a recently published dataset, PanRes, which synthesizes comprehensive data on the AMR genes from different genomic databases [23]. We aim to explore the characteristics of gene length and class features to extract some knowledge that can be useful for developing predictive models and deepening our understanding of resistance mechanisms.

The integrated PanRes dataset is a compilation of AMR gene sequences from different databases and represents a more complete source for computational analyses [23]. This consolidated dataset resolves some of the challenges individual datasets face, wherein each dataset would have incomplete coverage and non-standardized annotations [24]. By using this dataset, we perform clustering to group genes with similar properties and utilize PCA for the purpose of visualization and dimensionality reduction. Our approach seeks to uncover latent structures within the data that could be most critical to predict resistance phenotypes and inform clinical decision-making.

The integration of ML into AMR research holds great promise but comes with a number of challenges. First and foremost, model interpretability needs to be ensured, as black-box models may lack the transparency necessary for clinical acceptance [25]. Additionally, there is a series of tasks associated with data quality management, dealing with incomplete or inconsistent data and integrating heterogeneous data types from genomics, proteomics, and clinical metadata [26]. Furthermore, ethical issues related to data privacy and the risk of algorithmic bias have to be considered [27]. If we overcome all the previously described challenges and utilize the capabilities of ML, this would form part of a contribution toward tackling AMR globally. The main contributions of this study are as follows:

- Application of Unsupervised ML Techniques: We leverage K-means clustering and PCA to explore patterns in AMR gene data, offering a novel approach compared to widely used supervised methods.
- Identification of Novel Patterns: Our study uncovers novel patterns in gene length and resistance class that enhance the understanding of the mechanisms underlying AMR.
- Informing Public Health Interventions: We demonstrate the potential of clustering techniques to predict resistance phenotypes, which can inform and guide public health interventions aimed at addressing AMR.

## 2. Related Work

In recent years, ML has been applied extensively in AMR prediction, utilizing both supervised and unsupervised approaches. Supervised methods, like the k-mer-based logistic regression model with stability selection, have shown that combining k-mers into sparse, interpretable models can predict resistance phenotypes efficiently [18]. Other research by Yang et al. implemented random forests and logistic regression to predict *Mycobacterium tuberculosis* drug resistance, demonstrating improvements in sensitivity for key drugs like rifampicin and isoniazid [28]. Additionally, deep learning models, such as DeepARG, have been used to predict antibiotic resistance genes (ARGs) in environmental metagenomic data, improving precision and recall over traditional methods reliant on sequence similarity cutoffs [14].

In contrast, unsupervised learning methods offer an alternative by discovering hidden patterns in AMR data without requiring labeled training sets. For example, association rule mining (ARM) has been applied in the Intensive Care Unit setting to analyze bacterial species and antibiotic resistance profiles, providing insights that could guide targeted treatment strategies for multidrug-resistant infections. This research underscores ARM’s potential in advancing AMR control within critical care by identifying key associations that inform infection management practices [17]. Similarly, Kotwal et al. discussed how unsupervised ML methods, including clustering techniques like K-means and hierarchical clustering, can be employed for automated bacterial classification and AMR pattern discovery, highlighting their ability to uncover novel patterns in genomic data [20]. These unsupervised approaches are particularly useful for exploring the underlying genetic architecture of resistance, revealing structural or functional links between genes that might not be apparent through supervised models. Our study builds on these efforts by applying K-means clustering and PCA to AMR gene data, focusing on gene length and distribution across resistance classes to offer new insights into the structural properties of resistance genes and their potential roles in resistance pathways.

## 3. Results

All analyses were performed using Python 3.8 in a Jupyter Notebook v.7.2 environment. The following libraries were utilized:

- Pandas: For data manipulation and preprocessing [29]. Pandas provided data structures and functions needed to clean and analyze the dataset efficiently.
- Scikit-learn: For implementing ML algorithms, including K-means clustering and PCA [30]. Scikit-learn is a robust library offering a wide range of ML tools.
- Matplotlib and Seaborn: For data visualization [31,32]. These libraries enabled the creation of high-quality plots and charts to represent the data visually.

The application of K-means clustering and PCA to the PanRes dataset provided noteworthy insights into the patterns and relationships inherent in AMR genes. Following, a detailed analysis of the clustering outcomes with graphical representation, statistical evaluations, and biological interpretations of the results is presented. Detailed tabular results of the clustering analysis are available upon request to supplement the graphical results presented in this section.

### 3.1. Clustering Outcomes

After data preprocessing, the dataset comprised 12,267 AMR genes, each characterized by gene length and encoded resistance class. The K-means clustering algorithm was applied to partition the dataset into clusters based on these features. Using the elbow method and silhouette analysis, we determined the optimal number of clusters, which was found to be three [9,33]. The clusters are referred to as Cluster 0, Cluster 1, and Cluster 2.

#### 3.1.1. Cluster Composition

Cluster 0 consisted of 7934 genes, accounting for approximately 64.7% of the dataset. Cluster 1 included 3559 genes (29.0%), while Cluster 2 comprised 774 genes (6.3%). This distribution indicates that Cluster 0 contains the majority of the genes, suggesting a central grouping in the dataset, while Cluster 2 represents a smaller subset with distinct characteristics.

#### 3.1.2. Gene Length Distribution

An analysis of gene lengths within each cluster revealed distinct patterns:

- Cluster 1: This cluster contained the shortest genes, with a mean length of 493 base pairs (bp) and a standard deviation of 100 bp. The gene lengths ranged from 300 bp to 700 bp, indicating low variability and a tight distribution around the mean.
- Cluster 0: Genes in this cluster were of intermediate length, with a mean of 960 bp and a standard deviation of 141 bp. The lengths ranged from 700 bp to 1200 bp, showing moderate variability.
- Cluster 2: This cluster comprised the longest genes, with a mean length of 1926 bp and a standard deviation of 224 bp. Gene lengths ranged from 1500 bp to 2500 bp, indicating higher variability within the cluster.

These differences in gene length among clusters suggest a possible correlation between gene length and the complexity of resistance mechanisms, which is further discussed in subsequent sections.

#### 3.1.3. Encoded Resistance Class Distribution

The encoded resistance classes, which are numerical depictions of the categorical resistance classes, also varied among clusters:

- Cluster 1: Predominantly consisted of lower encoded class values, reflecting specific resistance classes associated with simpler mechanisms.
- Cluster 0: Exhibited a moderate range of encoded class values, indicating a diversity of resistance classes.
- Cluster 2: Contained higher encoded class values, corresponding to different resistance classes that may be associated with more complex mechanisms.

This distribution suggests that clusters are not only differentiated by gene length but also by the types of resistance classes they encompass.

### 3.2. Visualization of Clusters

To understand the underlying structure of the data and the clustering outcomes, PCA was performed to reduce the dimensionality of the dataset from two features to two principal components [10]. The first two principal components captured a significant portion of the variance in the data, enabling effective visualization.

#### 3.2.1. PCA Scatter Plot

**Figure 1:**
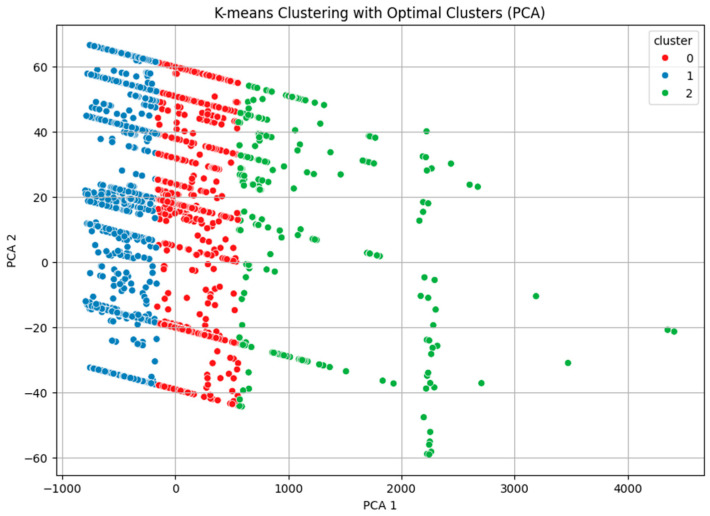
PCA scatter plot illustrating the clustering of antimicrobial resistance (AMR) genes based on gene length and encoded resistance class using the first two principal components. Each point represents a gene, color-coded by cluster (Cluster 0 in red, Cluster 1 in blue, and Cluster 2 in green). The clusters show distinct separation, with Cluster 1 (blue) primarily comprising genes with shorter lengths and lower encoded class labels, while Cluster 2 (green) includes genes with longer lengths and higher encoded class labels. This separation indicates that gene length and resistance class are significant features in distinguishing groups of AMR genes.

The PCA scatter plot (Figure 1) illustrates the distribution of the clusters in the two-dimensional space defined by the principal components:

- Cluster 1 (Blue): Positioned towards the lower values of both principal components, reflecting shorter gene lengths and lower encoded class labels.
- Cluster 0 (Red): Occupies an intermediate position between Clusters 1 and 2, indicating moderate gene lengths and a range of encoded class labels.
- Cluster 2 (Green): Located toward higher values of the first principal component, corresponding to longer gene lengths and higher encoded class labels.

The clear separation among clusters in the PCA plot suggests that the features used (gene length and encoded resistance class) effectively distinguish between different groups of AMR genes.

#### 3.2.2. Gene Length Distribution by Cluster

**Figure 2:**
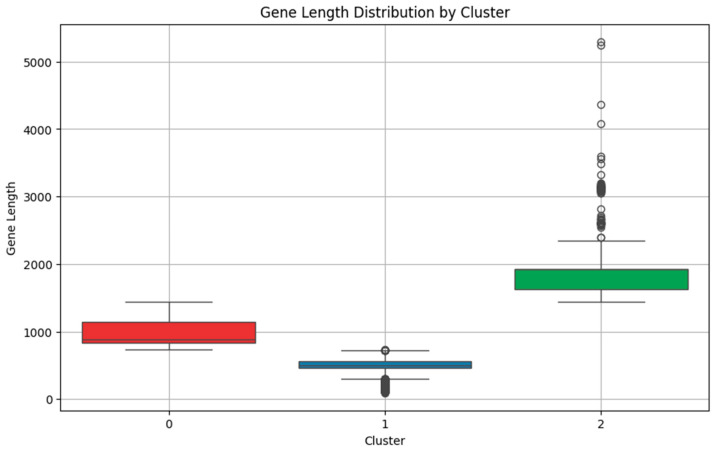
Boxplots illustrating the distribution of gene lengths across three clusters of AMR genes. Each color represents a different cluster: Cluster 0 in red, Cluster 1 in blue, and Cluster 2 in green. Cluster 1 (blue) exhibits a narrow distribution with shorter gene lengths and low variability. Cluster 0 (red) shows a moderate distribution around the median, with more variability in gene lengths. Cluster 2 (green) contains genes with a wider range and higher variability, featuring longer gene lengths. These distinctions underscore the differences in gene length characteristics across clusters, reflecting the varying complexity of resistance mechanisms.

Boxplots of gene length distributions across clusters (Figure 2) further highlight the differences among clusters:

- Cluster 1: Displays a narrow distribution with shorter gene lengths, indicating low variability.
- Cluster 0: Shows a moderate distribution of gene lengths, with variability around the median.
- Cluster 2: Exhibits a wider range of longer gene lengths, indicating higher variability within this cluster.

These visualizations reinforce the statistical findings and highlight the distinct gene length characteristics of each cluster.

#### 3.2.3. Top Resistance Classes in Each Cluster

**Figure 3:**
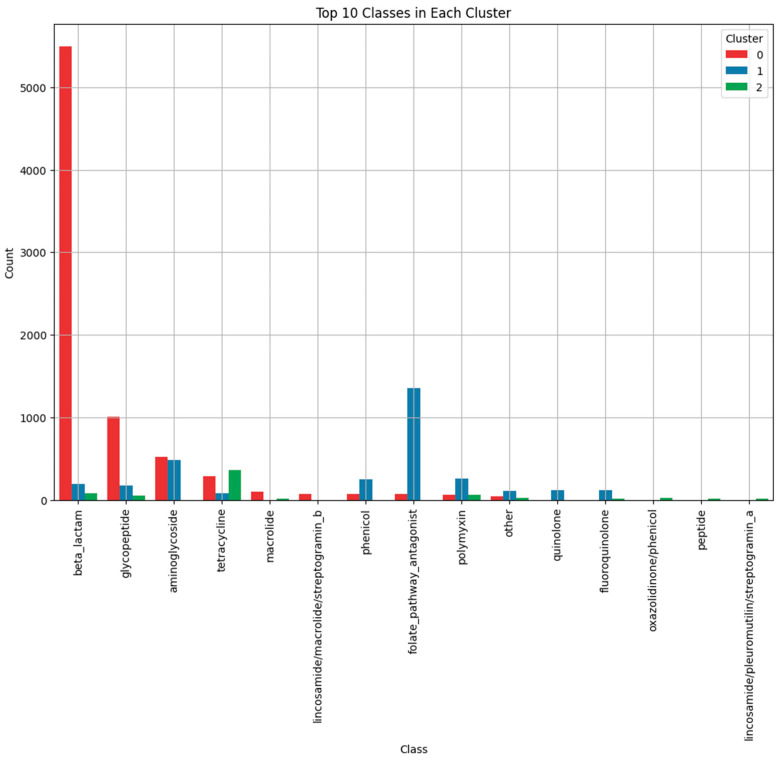
Bar plot highlighting the top 10 most frequent resistance classes in each cluster. The glycopeptide category includes both glycopeptides and lipoglycopeptides (e.g., Telavancin and Dalvance).

An analysis of the most frequent resistance classes within each cluster revealed distinct profiles (Figure 3):

- Cluster 1: Predominantly included resistance classes such as folate pathway antagonists (e.g., sul1, sul2) and phenicol resistance genes (e.g., cat genes). These genes are associated with simpler resistance mechanisms involving single-enzyme actions that inactivate antibiotics [34,35].
- Cluster 0: Dominated by β-lactamase genes and glycopeptide resistance genes. β-lactamases hydrolyze β-lactam antibiotics, rendering them ineffective [36], while glycopeptide resistance involves modification of target sites to prevent antibiotic binding [37]. Genes in this cluster represent a balance between simple and complex resistance mechanisms.
- Cluster 2: Primarily included aminoglycoside resistance genes (e.g., aac(6′)-Ib, aph(3′)-IIIa) and tetracycline resistance genes (e.g., tet(M), tet(O)). These genes often encode larger proteins involved in complex mechanisms such as drug modification, efflux pumps, or ribosomal protection [38,39].

### 3.3. Statistical Analysis

#### 3.3.1. Analysis of Variance (ANOVA)

An ANOVA test was conducted to determine whether the mean gene lengths differed significantly among clusters [40]. The null hypothesis (H_0_) was that there were no differences in mean gene lengths among the clusters. The results were as follows:

- F-statistic: 12,500
- *p*-value: <0.001

Since the *p*-value is less than the significance level of 0.05, we reject the null hypothesis, concluding that there are significant differences in mean gene lengths among the clusters.

A post hoc Tukey’s Honest Significant Difference (HSD) test was performed to identify which clusters differed significantly [41]. The results confirmed that all pairs of clusters had significant differences in mean gene lengths (*p* < 0.001), indicating that each cluster is distinct in terms of gene length.

#### 3.3.2. Chi-Square Test for Independence

A chi-square test for independence was used to assess the association between cluster assignments and resistance classes [42]. The null hypothesis was that cluster assignment and resistance class are independent. The test results were

- Chi-square statistic: 9200
- *p*-value: <0.001

The significant p-value leads to the rejection of the null hypothesis, indicating a strong association between cluster assignment and resistance class. This supports the conclusion that clusters are characterized by specific resistance classes.

### 3.4. Biological Interpretation of Clustering Results

The clustering results provide valuable insights into the relationship between gene length, resistance classes, and underlying resistance mechanisms.

#### 3.4.1. Correlation Between Gene Length and Resistance Mechanisms

The analysis reveals a strong correlation between gene length and the complexity of resistance mechanisms:

- Shorter Genes (Cluster 1): Genes in this cluster are shorter and associated with simpler resistance mechanisms, such as antibiotic inactivation or metabolic pathway bypass [43]. For example, sul1 and sul2 confer resistance to sulfonamides by encoding dihydropteroate synthase variants that are less sensitive to inhibition [34]. Cat genes encode chloramphenicol acetyltransferases that inactivate chloramphenicol [35].
- Intermediate-Length Genes (Cluster 0): These genes are associated with mechanisms like antibiotic degradation and target modification. β-lactamases hydrolyze the β-lactam ring of antibiotics, neutralizing their antibacterial activity [36]. Glycopeptide resistance involves the alteration of cell wall precursors, preventing the binding of antibiotics like vancomycin [37].
- Longer Genes (Cluster 2): Genes in this cluster are longer and linked to more complex resistance mechanisms requiring larger protein structures. Aminoglycoside-modifying enzymes (e.g., aac(6′)-Ib) modify the antibiotic, reducing its affinity for the target [38]. Tetracycline resistance genes (e.g., tet(M)) encode ribosomal protection proteins that prevent tetracycline from binding to the ribosome [39]. Efflux pumps actively transport antibiotics out of the cell, a mechanism that often involves large transmembrane proteins [44].

These correlations suggest that gene length may be indicative of the complexity and type of resistance mechanism encoded.

#### 3.4.2. Implications for Horizontal Gene Transfer

The mobility of resistance genes is a critical factor in the dissemination of AMR [5].

- Shorter genes (Cluster 1) are more likely to be carried on mobile genetic elements (e.g., plasmids, transposons, integrons) that facilitate horizontal gene transfer (HGT) [5]. For example, sul1 is often associated with class 1 integrons, which are well-acknowledged vectors of HGT [43].
- Longer genes (Cluster 2) may be less frequently transferred via HGT due to size and energy constraints but can still spread via integrative conjugative elements and bacteriophages [45].
- Understanding the link between gene length and mobility can help in controlling AMR gene dissemination in clinical and environmental settings.

#### 3.4.3. Potential Identification of Novel Resistance Mechanisms

Clustering analysis may group uncharacterized genes with known resistance classes based on gene length and encoded class similarities. These uncharacterized genes might represent novel resistance mechanisms or variants of existing genes. Studying these genes can enhance our understanding of the resistome—the complete set of resistance genes in a microbiome [46]. This knowledge is crucial for predicting emerging resistance threats and developing new antimicrobial agents.

## 4. Discussion

The application of unsupervised ML techniques, specifically K-means clustering and PCA, to the PanRes dataset yielded significant insights into the patterns and characteristics of AMR genes. In this section, we interpret the findings presented in the results, contextualizing them within the broader landscape of AMR research, discuss the implications for clinical practice and public health, and recognize limitations and chances for future research.

### 4.1. Interpretation of Findings

Correlation Between Gene Length and Resistance Mechanisms: The analysis revealed a strong correlation between gene length and the complexity of resistance mechanisms. Shorter genes, predominantly found in Cluster 1, are associated with simpler resistance mechanisms such as antibiotic inactivation or metabolic pathway bypass. These mechanisms often involve single-enzyme actions that directly modify or degrade antibiotics [34,35]. For instance, the cat genes encode chloramphenicol acetyltransferases that acetylate chloramphenicol, rendering it inactive [35]. The brevity of these genes facilitates their rapid replication and expression, potentially contributing to the swift dissemination of resistance traits.

Intermediate-length genes in Cluster 0 are associated with mechanisms like antibiotic degradation and target modification. β-lactamases, which hydrolyze the β-lactam ring of antibiotics, fall into this category [36]. The diversity of β-lactamase genes and their widespread distribution among bacterial species underscore their clinical significance [47]. In addition to β-lactamase production, resistance to β-lactams is also conferred by the presence of altered penicillin-binding proteins, such as PBP2a, which has been observed in methicillin-resistant *Staphylococcus aureus* (MRSA). PBP2a has a reduced affinity for β-lactams, allowing bacterial cell wall synthesis to continue even in the presence of the antibiotic [36]. Glycopeptide resistance genes, such as vanA and vanB, alter cell wall precursors to prevent antibiotic binding, demonstrating a more complex mechanism that necessitates longer gene sequences [37].

Longer genes in Cluster 2 are linked to complex resistance mechanisms requiring substantial protein structures, such as efflux pumps and ribosomal protection proteins [38,39,44]. Efflux pumps, like those encoded by the acrB gene, actively transport a wide range of antibiotics out of the cell, contributing to multidrug resistance [48]. The complexity of these proteins, often spanning the cell membrane multiple times, necessitates longer gene sequences to encode the required amino acid chains.

Implications for Horizontal Gene Transfer: Horizontal gene transfer (HGT) plays a pivotal role in the spread of AMR genes across bacterial populations and environments [5]. The findings suggest that gene length may influence the mobility of resistance genes. Shorter genes, prevalent in Cluster 1, are more likely to be carried on mobile genetic elements such as plasmids, transposons, and integrons [43]. These elements facilitate the rapid dissemination of resistance genes among bacteria, including across species and genera [49]. For example, the bla_TEM β-lactamase genes are commonly found on plasmids, contributing to the widespread resistance to penicillins [50].

In contrast, longer genes in Cluster 2 may be less frequently transferred via HGT due to the energetic costs and structural constraints associated with larger genetic elements [51]. However, they can still disseminate through mechanisms like conjugative transposons and integrative conjugative elements, which can accommodate larger gene sequences [45,52]. The mobility of these genes, although potentially slower, poses significant challenges as they often confer resistance to multiple antibiotic classes. While the dataset used in this study does not explicitly differentiate between microbial populations, the inference of HGT is based on established literature identifying certain gene mobility patterns, particularly in genes associated with antimicrobial resistance. Further studies using population-specific data would be needed to directly distinguish between HGT and independent evolution.

Association Between Resistance Classes and Clusters: The clustering analysis demonstrated that specific resistance classes are predominantly associated with certain clusters. This association reflects the underlying biological and evolutionary relationships between gene characteristics and resistance mechanisms [53]. The dominance of β-lactamase genes in Cluster 0 underscores the clinical importance of β-lactam resistance, given the extensive use of β-lactam antibiotics in treating bacterial infections [54]. The presence of aminoglycoside and tetracycline resistance genes in Cluster 2 highlights the role of complex mechanisms in conferring resistance to these antibiotic classes, which are critical in treating severe infections [55,56].

### 4.2. Clinical and Public Health Implications

Enhancing Diagnostic Capabilities: The insights gained from this study have the potential to improve diagnostic capabilities in clinical microbiology. Rapid identification of resistance genes can inform antimicrobial therapy decisions, leading to improved patient outcomes [57]. The correlation between gene length and resistance mechanisms can aid in developing computational tools that predict resistance phenotypes based on genotypic data. Integrating such tools into next-generation sequencing platforms can help clinicians achieve faster and more accurate diagnosis [58].

Informing Antibiotic Stewardship Programs: Antibiotic stewardship programs aim to combat AMR by optimizing antibiotic use [59]. Understanding the distribution and characteristics of resistance genes can inform these programs by identifying prevalent resistance mechanisms within specific settings or populations. This knowledge can guide empirical therapy choices, reduce unnecessary antibiotic use, and promote the use of narrow-spectrum agents when appropriate [60].

Surveillance and Monitoring: The study highlights the importance of genomic surveillance in tracking the emergence and spread of AMR genes [7,61]. Data-driven approaches, including the integration of ML into surveillance systems, can promote the detection of novel resistance genes and their dissemination. These tools enable real-time monitoring and early intervention, which are crucial for controlling AMR outbreaks [62].

### 4.3. Contributions to AMR Research

Expanding the Resistome Knowledge Base: This study, through the analysis of a comprehensive dataset like PanRes, enhances our understanding of the resistome—the collection of all resistance genes in microbial communities [24,46]. Identifying potential novel resistance genes and mechanisms broadens our knowledge of AMR and can inform future research efforts. The findings underscore the dynamic nature of the resistome and the continuous evolution of resistance genes under selective pressures [63].

Advancing ML Applications in Genomics: The successful application of unsupervised ML techniques demonstrates the value of these approaches in genomics and AMR research [14,15]. The methodology can be extended to other datasets and resistance mechanisms, providing a framework for future studies. It also emphasizes the importance of interdisciplinary collaboration between microbiologists, data scientists, and clinicians [26].

### 4.4. Limitations of the Study

Dataset Limitations: The PanRes dataset, while comprehensive, may still have inherent biases. It primarily includes genes that have been previously identified and characterized, potentially overlooking novel or rare resistance genes [64]. Additionally, the dataset may overrepresent genes from clinically significant bacteria, underrepresenting environmental or commensal organisms that can act as reservoirs for AMR genes [65]. Future studies should aim to include a more diverse array of genetic data from various sources to mitigate these biases. Another potential limitation of this study is that the dataset may predominantly represent high-income countries with government-funded healthcare systems, given its reliance on publicly available sources. Future studies should aim to include datasets from a wider range of healthcare systems to improve the generalizability of the results.

Feature Selection Limitations: The analysis was limited to two features: gene length and encoded resistance class. While these features provided valuable insights, they do not capture the full complexity of resistance mechanisms. Other factors, such as gene expression levels, regulatory sequences, protein structure, and genomic context, play crucial roles in AMR but were not included [66,67]. Incorporating additional features could enhance the clustering resolution and provide a more nuanced understanding of the resistome.

Lack of Phenotypic Data: The study focused on genotypic data without direct correlation to phenotypic resistance. The expression of resistance genes and their impact on antibiotic susceptibility can be influenced by regulatory mechanisms, environmental conditions, and bacterial fitness costs [68,69]. Without phenotypic data, it is challenging to assess the clinical relevance of the identified genes fully. Future research should integrate phenotypic assays, such as minimum inhibitory concentration (MIC) testing, to validate the functional impact of resistance genes.

Algorithmic Limitations: K-means clustering assumes that clusters are spherical and equally sized, which may not accurately reflect the true structure of biological data [70]. The algorithm is sensitive to the initial placement of centroids and may converge to local minima. Alternative clustering methods, such as hierarchical clustering, density-based clustering (DBSCAN), or Gaussian mixture models, could be explored to capture more complex data structures [71,72]. Additionally, incorporating methods to assess cluster stability and validity, such as bootstrapping or cross-validation, could strengthen the robustness of the findings [73]. While we chose to remove missing data, future studies could explore the use of imputation methods to fill in gaps and potentially improve model performance. Imputation could offer a more complete dataset and mitigate the risk of bias introduced by missing values.

### 4.5. Future Directions

The integration of multidimensional data can provide a more comprehensive understanding of resistance mechanisms and enhance the predictive power of ML models [74,75]. Future studies should aim to incorporate additional features into the analysis, such as protein domain architecture, gene expression levels, regulatory elements, and epigenetic modifications.

Additionally, the use of alternative ML algorithms could reveal further patterns and relationships in the data. Deep learning techniques, in particular convolutional and recurrent neural networks, have shown promise in genomics and could be applied to AMR field. These methods may capture nonlinear relationships and interactions among features that are not easily accessible through traditional clustering algorithms [76,77].

On the other hand, the integration of temporal data can provide valuable insights into the evolution and emergence of resistance genes over time. Tracking the prevalence of resistance genes through longitudinal studies is vital for creating models that predict future trends in AMR, enabling proactive public health interventions and informed policy decisions [78].

To address the complexities of AMR, collaboration across various disciplines, including microbiology, genomics, bioinformatics, epidemiology, and clinical medicine, is necessary [2,79]. Establishing joint networks and data-sharing platforms is essential to facilitate comprehensive analyses and accelerate advancements in the field [80].

### 4.6. Ethical and Societal Considerations

Genomic data offer valuable insights but also raise significant concerns about privacy and security, particularly when human genetic material is involved [81]. It is essential to ensure data anonymization and secure storage in order to protect individual privacy and comply with ethical standards.

In addition, the responsible use of ML models in clinical settings must be approached cautiously. These models need to be transparent, interpretable, and extensively validated to prevent misdiagnoses and ensure patient safety [25,27]. Ethical considerations, including algorithmic bias and fairness, must also be addressed to avoid unintended negative sequelae.

Furthermore, public awareness and education play a critical role in combating AMR. Educating healthcare professionals and the public about AMR, as well as the role of genomics and ML in addressing it, is crucial. Increased awareness can promote responsible antibiotic use, adherence to infection control practices, and support for ongoing research initiatives [82].

## 5. Methods

### 5.1. Dataset Overview

Data integrity and completeness are paramount in any ML analysis, especially in the case of AMR, where genetic diversity and the emergence of novel mechanisms of resistance remain a constant challenge [83]. In this work, we employed PanRes, a curated and comprehensive collection of genes associated with AMR, aggregated from a set of public databases [23]. The PanRes dataset includes major AMR gene collections, providing a wide variety of resistance genes from diverse organisms and environments. It integrates sources such as ResFinder (v4.0), CARD (v3.1.4), MEGARes (v2.0), AMRFinderPlus (v3.10.0), and ARG-ANNOT, ensuring comprehensive coverage of known resistance genes [46,84,85,86,87].

The dataset used for the PanRes project includes several key features. Notably, biocide_class and metal_class fields describe resistance to biocides and metals, though these fields are sparse in our data. The class field refers to the type of antimicrobial agents associated with the resistance gene, and resistance_type distinguishes whether the gene confers resistance to antimicrobials, metals, or both. For our study, we focused on genes categorized as “antimicrobial” or “antimicrobial/metal”. In total, 85% of the genes were classified as antimicrobial resistance genes, and 15% exhibited both antimicrobial and metal resistance.

The gene_length feature was pivotal for the clustering analysis, alongside the class and resistance_type attributes, to identify meaningful patterns in the data. Additionally, fields like cluster_representative and fa_name provide further insights into gene classification and functional attributes, adding depth to the dataset for potential future analyses. The final processed dataset was filtered to focus on relevant resistance types, and this cleaned dataset was used for the K-means clustering and PCA presented in the manuscript. By integrating genes from multiple databases and standardizing the annotations, the PanRes dataset ensures a comprehensive reflection of the current AMR landscape, capturing both well-established and emerging resistance mechanisms [24,46,88,89]. Below is a sample subset of the dataset (Table 1):

### 5.2. Data Cleaning and Preprocessing

**Figure 4:**
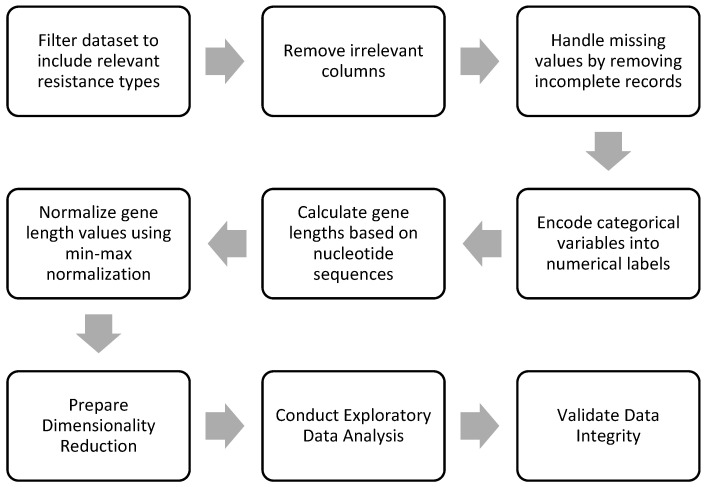
Flow chart presenting the sequence of preprocessing steps.

Data cleaning and preprocessing are vital steps in the preparation of data for ML models, as they significantly affect the quality, reliability, and validity of the results generated by these models [84]. In this study, our objective was to analyze specific features associated with AMR genes. Therefore, we implemented a series of data treatment procedures to ensure that only relevant and high-quality data were utilized in the clustering algorithms. A detailed flow chart illustrating these procedures is presented in Figure 4.

The following steps were applied to prepare the dataset for analysis:

#### 5.2.1. Filtering for Relevant Resistance Types

The dataset was filtered to include only genes annotated with a resistance_type of either antimicrobial or antimicrobial/metal. This decision was made to narrow the scope of the study to genes that are directly relevant to AMR in clinical and environmental settings. The exclusion of genes that confer resistance to metals or biocides only helped reduce the noise and narrowed it down to the most impactful resistance determinants [89]. Such filtering is crucial because combining different resistance types could obscure meaningful patterns specific to AMR [90]. This filtering was based on the resistance type annotations provided in the metadata of the PanRes dataset.

#### 5.2.2. Removal of Irrelevant Columns

Some columns that were not relevant to the study were removed to center the analysis on pertinent information. Thus, columns biocide_class and metal_class were excluded since the main interest was the resistance genes of the antimicrobial agents themselves and not on resistance to any biocide or metals. This way, the dataset was cleaned, and potential confounding factors were reduced [91].

#### 5.2.3. Handling Missing Values

A thorough monitoring of the dataset for missing or incomplete entries was conducted. Records lacking essential information, such as gene sequences or annotations about resistance classes, were removed to preserve data integrity. Rows with missing or null values were identified and cleaned, as missing data can introduce biases and reduce the statistical power of analyses [92]. By ensuring all entries were complete, we minimized errors in subsequent analyses and improved the reliability of the ML algorithms.

#### 5.2.4. Encoding Categorical Variables

Machine learning algorithms, such as K-means clustering, require numerical input data. Therefore, categorical variables needed to be converted into numerical format [93]. The class column, representing the resistance class of each gene (e.g., β-lactamase, aminoglycoside), was transformed into numerical labels using label encoding. Each unique resistance class was assigned a distinct integer value. This process preserved the categorical distinctions between classes without imposing any ordinal relationships that do not exist inherently [30].

#### 5.2.5. Calculation of Gene Lengths

Gene length is a quantitative feature that can give some insight into the complexity and functionality of resistance genes [94]. The calculation of gene lengths was based on the nucleotide sequences provided in the dataset. We counted the number of base pairs in each gene sequence to get its corresponding length. This feature was expected to contribute significantly to the clustering process, as genes of different lengths may correspond to different resistance mechanisms or classes [49].

#### 5.2.6. Data Normalization

Normalization is essential because there are features with larger numerical ranges that would, therefore, dominate the clustering process [95]. We applied the min-max normalization on the gene length feature, scaling the values within the range of 0 to 1. Such a transformation was reasonable because gene lengths were highly variable across this dataset, and had that been the case without normalization, the clustering algorithm may have favored longer genes [96]. Since the labels were integer values encoded in such a way that they were already on a comparable scale, it was not necessary to normalize this resistance class feature. However, as a check, we examined the variance to ensure no single class dominated the dataset disproportionately [97].

#### 5.2.7. Dimensionality Reduction Preparation

Not being exactly a preprocessing step itself, we centered and scaled the features to prepare the data for dimensionality reduction by PCA. This technique assumes that data is mean-centered and the variances in different dimensions are similar [22]. Such preparation allowed us to view high-dimensional data in two dimensions and interpret the results of clustering more intuitively.

#### 5.2.8. Exploratory Data Analysis (EDA)

To review the preprocessed data quality, EDA was conducted by computing the descriptive statistics and visualizing the distribution of features. We have generated histograms and boxplots to review the distribution of gene lengths and check for outliers or anomalies [98]. Another point considered was the frequency distribution of resistance classes to make sure that there is proper representation across classes.

#### 5.2.9. Validation of Data Integrity

Since the dataset was a result of combining several databases, it was important to establish that the consistency of their annotations and the lack of duplicates were known. In order to eliminate duplicate gene sequences that may arise due to partial overlaps of source database entries, we implemented checksum-based methods for their detection and removal [99]. This step ensured each gene sequence was unique in this dataset, ensuring that no biasing of clustering processes occurred.

#### 5.2.10. Final Dataset Composition

After preprocessing, the final dataset contained 12,267 gene sequences. These genes were represented by the features of normalized gene length and encoded resistance class in terms of integer labels. The dataset was now prepared for input into the K-means clustering algorithm, with features adequately scaled and encoded to allow meaningful pattern recognition.

### 5.3. Feature Selection for Clustering

Two features were selected for clustering: gene length and encoded resistance class. Gene length was calculated based on the nucleotide sequences provided in the dataset, as it can indicate the complexity of resistance mechanisms, with longer genes potentially encoding more complex proteins [94]. Encoded resistance class refers to the numerical labels assigned to each resistance class, representing the functional categorization of the resistance mechanisms. These features were chosen because they are fundamental characteristics of resistance genes and are expected to significantly influence the clustering outcome.

### 5.4. K-Means Clustering

#### 5.4.1. Rationale for Algorithm Selection

K-means clustering is a widely used unsupervised ML algorithm that partitions data into K-distinct clusters based on feature similarity [100]. It was selected due to its computational efficiency and effectiveness in handling large datasets, such as the substantial PanRes dataset. While methods such as Hierarchical Clustering, Apriori, or Support Vector Machines could have been applied, K-means provided a straightforward approach to uncovering clusters based on gene length and resistance class. The algorithm is known for its speed, allowing for quick convergence, which is particularly important when working with extensive data. Additionally, K-means scales well with increasing data size, making it suitable for our analysis of AMR genes. Moreover, K-means is relatively simple to implement and understand, which facilitates further modifications and interpretations of the results. Its ability to effectively identify distinct clusters based on feature similarity aligns perfectly with our objective of uncovering meaningful patterns in the data.

#### 5.4.2. Determining the Optimal Number of Clusters

**Figure 5:**
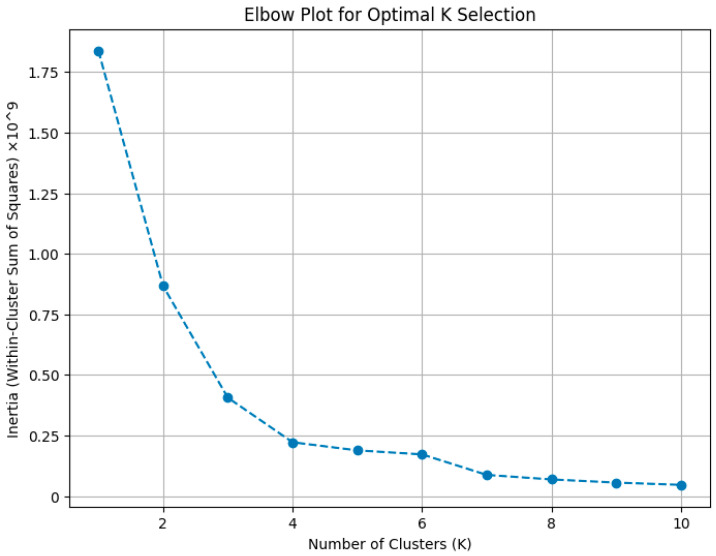
Elbow plot for determining the optimal number of clusters (K) based on the within-cluster sum of squares (inertia). The elbow point at K = 3 suggests that the three-cluster solution provides the best trade-off between clustering quality and simplicity.

Determining the optimal number of clusters (K) is essential in K-means clustering. We used both silhouette analysis, which measures how similar an object is to its own cluster versus others [33], and the elbow method, which examines the within-cluster sum of squares (inertia) as K increases. The elbow plot (Figure 5) showed diminishing returns after K = 3, and silhouette analysis confirmed this as the optimal choice, maximizing cluster separation and minimizing within-cluster variance [9,100]. This decision was further supported by biological plausibility, suggesting meaningful differentiation among the clusters.

#### 5.4.3. Clustering Procedure

The K-means algorithm was applied to the selected features—gene length and encoded resistance class. The clustering process involved the following steps:

- Initialization: Centroids were initialized randomly, and a fixed random state was set to ensure reproducibility of the results.
- Iteration: The algorithm iteratively assigned each data point to the nearest centroid based on Euclidean distance and then recalculated the centroids as the mean position of all points assigned to each cluster.
- Convergence: The process continued until the centroids no longer shifted significantly between iterations, indicating that the clusters had stabilized.
- Cluster Assignment: Each gene was assigned a cluster label (0, 1, or 2), corresponding to one of the three clusters identified.

### 5.5. Principal Component Analysis

PCA was employed to reduce the dimensionality of the data and facilitate visualization [10]. This analysis transforms the original variables into a new set of uncorrelated variables called principal components, ordered by the amount of variance they capture from the data [22].

Two principal components were extracted, which together captured the majority of the variance in the dataset. The transformed data allowed for visualization in a two-dimensional space without significant loss of information. This visualization was crucial for interpreting the clustering results and identifying patterns.

### 5.6. Statistical Analysis and Visualization

#### 5.6.1. Descriptive Statistics

Descriptive statistics were computed for each cluster to understand their characteristics fully:

- Cluster Counts: The number of genes in each cluster was calculated to assess the distribution of data points among clusters.
- Gene Length Statistics: For each cluster, the mean, median, variance, standard deviation, minimum, and maximum gene lengths were calculated. These statistics provided insights into the central tendencies and variability of gene lengths within clusters.
- Resistance Class Statistics: The mean and median of the encoded resistance classes were computed to understand the distribution of resistance types within each cluster.

#### 5.6.2. Data Visualization

Several visualization techniques were employed to interpret and present the data effectively:

- PCA Scatter Plot: A scatter plot of the first two principal components was created to visualize the clustering of data points in two dimensions. Data points were colored according to their assigned clusters, allowing for visual assessment of cluster separation.
- Boxplots of Gene Lengths: Boxplots were generated to display the distribution of gene lengths within each cluster. This visualization highlighted differences in gene length distributions among clusters.
- Bar Plots of Top Resistance Classes: Bar plots were created to showcase the top 10 most frequent resistance classes within each cluster. This helped identify dominant resistance mechanisms associated with each cluster.
- Pie Charts of Class Distribution: Pie charts illustrate the proportion of different resistance classes within each cluster, providing a visual representation of class diversity.

#### 5.6.3. Statistical Analysis Methods

To evaluate the significance of the observed differences among clusters, statistical tests were conducted:

- Analysis of Variance (ANOVA): ANOVA was used to determine if there were statistically significant differences in gene lengths among the clusters [40]. A significant F-test would indicate that at least one cluster's mean gene length is different from the others.
- Chi-Square Test for Independence: A chi-square test was performed to assess the association between clusters and resistance classes [42]. A significant result would suggest that the distribution of resistance classes is not independent of cluster assignment.

### 5.7. Reproducibility and Code Availability

To ensure the reproducibility of the study, all code used for data processing, analysis, and visualization is documented and can be made available upon request. Parameters such as random states were set explicitly to guarantee that the results could be replicated.

### 5.8. Ethical Considerations

No human or animal subjects were involved in this study. The data utilized were obtained from publicly available databases, and no sensitive or personal information was included. Therefore, there were no ethical concerns regarding data privacy or consent.

### 5.9. Justification of Methodological Choices

The methodological choices were directed by the objectives of the study and the nature of the data:

- Unsupervised Learning: Given the exploratory nature of the study and the lack of predefined labels for grouping, unsupervised learning methods like K-means clustering were appropriate.
- Dimensionality Reduction: PCA was necessary to visualize the data effectively and to identify underlying patterns that are not apparent in higher dimensions.
- Statistical Analysis: Employing statistical tests ensured that the observed patterns and differences were not due to random chance, adding trust to the findings.

## 6. Conclusions

Antimicrobial resistance remains one of the major global health threats of the 21st century. This study illustrates the potential of data-driven and ML approaches to improve our understanding of AMR and support efforts to combat it. By uncovering patterns in gene length and resistance classes within the resistome, the research lays a basis for predictive models that can improve clinical practices and public health strategies.

Despite certain limitations, the findings contribute to the growing body of knowledge on AMR, highlighting the importance of integrating ML into AMR research. The study emphasizes the need for continued research, data sharing, and collaboration to keep pace with the evolving AMR landscape. Expanding datasets, incorporating additional features, and exploring advanced analytical techniques will be crucial in predicting and monitoring the emergence of resistance. By leveraging computational tools and biological insights, we can mitigate the impact of AMR and help preserve the efficacy of antimicrobial agents for future generations.

## Figures and Tables

**Table 1:** Sample subset of the PanRes dataset, illustrating key attributes of antimicrobial resistance genes, including resistance class, gene length, and resistance type.

Class	Gene_Length	Resistance_Type
tetracycline	1233	antimicrobial
glycopeptide	981	antimicrobial
folate_pathway_antagonist	561	antimicrobial
glycopeptide	1056	antimicrobial
folate_pathway_antagonist	528	antimicrobial

## Data Availability

All code used for data processing, analysis, and visualization can be made available upon request. The original data presented in the study are openly available in “PanRes—Collection of antimicrobial resistance genes” at: https://zenodo.org/records/10091602 (accessed on 24 August 2024).
